# The EFP Formation and Penetration Capability of Double-Layer Shaped Charge with Wave Shaper

**DOI:** 10.3390/ma13204519

**Published:** 2020-10-12

**Authors:** Yakun Liu, Jianping Yin, Zhijun Wang, Xuepeng Zhang, Guangjian Bi

**Affiliations:** School of Mechatronic Engineering, North University of China, Taiyuan 030051, China; lykcpy@163.com (Y.L.); wzj@nuc.edu.cn (Z.W.); zhangxp@nuc.edu.cn (X.Z.); bgjnuc@163.com (G.B.)

**Keywords:** wave shaper, double-layer shaped charge, explosively formed projectile, overdriven detonation, penetration

## Abstract

Detonation waves will bypass a wave shaper and propagate in the form of a horn wave in shaped charge. Horn waves can reduce the incidence angle of a detonation wave on a liner surface and collide with each other at the charge axis to form overdriven detonation. Detection electronic components of small-caliber terminal sensitive projectile that are limited by space are often placed inside a wave shaper, which will cause the wave shaper to no longer be uniform and dense, and weaken the ability to adjust detonation waves. In this article, we design a double-layer shaped charge (DLSC) with a high-detonation-velocity explosive in the outer layer and low-detonation-velocity explosive in the inner layer. Numerical and experimental simulation are combined to compare and analyze the forming process and penetration performance of explosively formed projectile (EFP) in DLSC and ordinary shaped charge (OSC). The results show that, compared with OSC, DLSC can also adjust and optimize the shape of the detonation wave when the wave shaper performance is poor. DLSC can obtain long rod EFPs with a large length-diameter ratio, which greatly improves the penetration performance of EFP.

## 1. Introduction

In a small-caliber terminal sensitive projectile, the warhead needs a set of detection and guidance electronic components to control flight of the projectile. If these electronic components are placed in the front of the warhead, it will affect the forming of the liner to a certain extent and reduce the performance of the shaped charge damage element. Therefore, these electronic components are generally placed inside wave shaper, which can not only solve the placement problem of electronic components, but also optimize the structure of detonation waves. However, placing electronic components inside a wave shaper will cause the wave shaper to no longer be uniform and dense, and the ability to adjust detonation waves will decrease. Shaped charges usually use high-energy sensitive explosives. After the performance of the wave shaper is reduced, the wave shaper must have enough thickness to block the propagation of detonation waves at the axis, which conflicts with the limited space of shaped charge. The outer layer of a double-layer shaped charge (DLSC) adopts high-detonation-velocity explosive (HE) and the inner layer adopts low-detonation-velocity explosive (LE). When the performance of wave shaper is poor, detonation waves in DLSC can also propagate in the form of horn waves, which reduce the incidence angle of the detonation wave on liner surface, and then collide with each other at the axis to form overdriven detonation. Overdriven detonation wave formed in OSC will eventually decay into a steady spherical wave propagation, while the axis of DLSC can always remain in the overdriven detonation state [1,2].

Explosively formed projectiles (EFP) tend to develop in the direction of more stable flight, higher accuracy, and stronger penetration ability, so as to strike more solid targets at a greater distance. This requires EFP to have a better aerodynamic shape and a larger length-diameter ratio. Li [3,4] used the method of multi-point initiation and used the collision of detonation waves to generate overdriven detonation, and obtained a long rod type EFP. Weimann [5] obtained a large length-diameter ratio EFP with empennage by embedding wave shaper in shaped charge. Zhu [6] studied three kinds of detonation waveforms in shaped charge with wave shaper (SCWS), and obtained a method to avoid the fracture of EFP with large length-diameter ratio by optimizing the structure of wave shaper. Many scholars carried out numerical simulation researches of shaped charges [7,8,9,10,11,12]. By comparing with the test results, the most appropriate material parameters and state equations are adopted to obtain influence laws of various factors on the formation process of shaped charges. Cardoso [13] conducted the research of EFP formation process and velocity attenuation. Liu [14] used the grey system theory to analyze the influence laws of the related parameters on the formation of EFPs. In addition, many new materials have been applied to the liner of shaped charges [15,16,17,18], which improves the penetration capability and damage aftereffect of shaped charges. Müller [19] proved through experimental research that detonation waves in SCWS collide with each other and meet certain conditions that will form a Mach wave and produce overdriven detonation. Dunne [20,21] obtained the calculation method of the Mach wave parameters, and pointed out that the polytropic exponent of explosives determines the critical incidence angle when detonation waves collide with each other to form Mach waves. Pan [22,23] carried out a more in-depth study on the propagation and collision process of detonation waves in SCWS.

Regarding the research of DLSCs, Held [24] designed a kind of DLSC, the LE is TNT (trinitrotoluene), glass and HMX (cyclo tetramethylene tetra nitramine)/adhesive (85/15), the HE is TNT/HMX (15/85). He used a striped mirror camera to observe the propagation process of detonation waves in the DLSC and confirmed that convergent horn waves can be formed in the DLSC. Zhang [25] and Wang [26] carried out experimental research about the formation and penetration of jets in DLSC. In their numerical simulations, Lee-Tarver equation was used to describe the overdriven detonation process in DLSC. The results show that compared with OSC, the head-velocity of jet in DLSC is increased by about 30%. Liu [27] established the propagation path mathematical model of detonation waves in DLSC and pointed out that the overdriven detonation intensity in DLSC is determined by polytropic exponent and detonation velocity of explosives. In addition, Skrzypkowski [28] analyzed the impact of detonation waves on the bearing capacity of anchor shells in ore mining.

Combining the above background, this article applies DLSC to the EFP warhead and compares the propagation form of detonation waves in OSC and DLSC. The forming process and penetration ability of EFP under different shaped charge structures are comparatively compared. Numerical simulation and experimental results are consistent. The results show that DLSC can greatly improve the penetration capability of EFP compared with OSC. The research results can provide a new idea for the structure design of small-caliber terminal sensitive projectile.

## 2. Detonation Wave Propagation Path in DLSC

**Figure 1:**
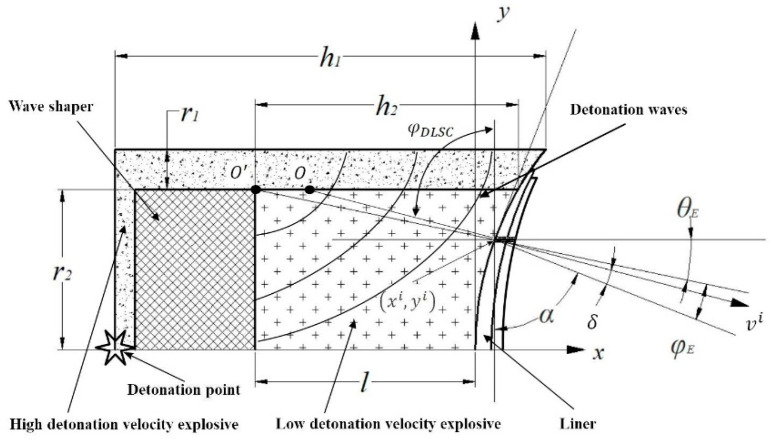
Detonation wave propagation in DLSC.

As shown in Figure 1, the detonation point is on the left side of wave shaper. The detonation wave is diffracted through the wave shaper and propagated in the form of horn waves in DLSC. The incident angle of detonation waves on liner surface is $\varphi_{E}$, that is, the angle between the propagation direction of detonation wave and the normal direction of liner surface. As known from the Huygens principle, every point on primary waves can be regarded as a new wave source of secondary waves. The velocity and frequency of secondary waves are equal to the primary waves, and the envelope surface formed by secondary waves is the new wave surface of primary waves at this moment. In OSC, detonation wave propagates at the center point $O^{\prime }$ after being diffracted by wave shaper. In DLSC, the center point of the detonation wave after diffraction changes continuously with increasing propagation distance.

According to our previous researches [2,27], in a DLSC, detonation waves that propagate in the LE are no longer centered at the $O^{\prime }$ point and the center of the detonation waves becomes the O point. The incident angle of detonation wave in the horizontal direction of shaped charge is $\varphi_{DLSC}$,

(1) $$\varphi_{DLSC}=arcsin\left\{\frac{\left[n^{2}+{\left(D_{L}r^{i}\right)}^{2}-2D_{H-L}n\sqrt{l^{2}+r^{i^{2}}}+2D_{L}ln\right]}{{\left(D_{L}r^{i}\right)}^{2}+n^{2}}\right\}$$

in the Equation (1), $n=D_{H-L}\sqrt{l^{2}+r^{i^{2}}}-D_{L}l$, $r^{i}=r_{2}-y^{i}$. $D_{L}$ is the detonation velocity of LE, $D_{H-L}$ is the propagation velocity of detonation wave when the LE is shocked initiation by the HE near the contact surface of two explosives. u is the particle velocity of explosive. The subscripts H and L represent the HE and LE respectively.

(2) $$D_{H-L}=D_{L}\left(\frac{u_{H}}{u_{L}}+\frac{u_{L}}{u_{H}}\right)/2,\;u=\frac{1}{k+1}D$$

From the geometric relationship in Figure 1, the value of $\varphi_{E}$ can be obtained.

(3) $$\{\begin{array}{c} \varphi_{E}=\varphi_{DLSC}-\alpha ;y^{i}<r_{2}\;\\ \varphi_{E}=90-\alpha +\theta ,\theta =arctan\left(\frac{y^{i}-r_{2}}{x^{i}+l}\right);y^{i}>r_{2} \end{array}$$

where α is the equivalent half cone angle. φ is the incidence angle of detonation waves on the surface of micro-elements. The micro-element velocity direction is not perpendicular to the surface of micro-elements, but has a certain angle with the normal direction of the surface and the angle is δ. $\theta_{E}$ is the angle between the propagation direction of detonation waves and the horizontal direction. $r_{1}$ is the thickness of the high-detonation-velocity explosive, $r_{2}$ is the radius of the wave shaper, $h_{1}$ is the length of the shaped charge, $h_{2}$ is the length of the low-detonation-velocity explosive, l is the distance between the top of the liner and the wave shaper,

(4) $$\alpha =arctan\left[f^{\prime }\left(x^{i}\right)\right]$$

It can be seen from the above equations that compared with an OSC, a DLSC can reduce the incident angle of detonation waves on the liner surface and increase the initial pressure. The greater the difference in detonation velocity between the inner and outer layers of DLSC, the more significant the adjustment to detonation waves.

## 3. Numerical Simulation

**Figure 2:**
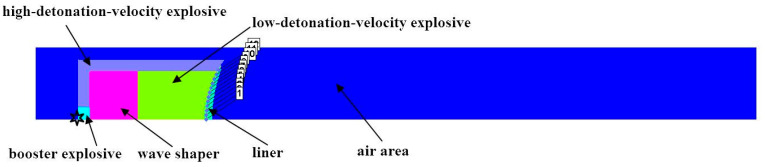
Finite-element numerical calculation model.

In this article, we used the nonlinear dynamics software Ansys-Autodyn to numerically simulate the formation and penetration of EFPs. In the numerical calculation, the asymmetry of the shaped charge caused by the manufacturing and assembly process is ignored, and established a two-dimensional axisymmetric calculation model. The Lee-Tarver equation of state is used in the overdriven detonation process of explosives [1,27]. Since some parameters of the Lee-Tarver equation are fitted in the (cm, g, μs) unit system, so the calculation model adopts the (cm, g, μs) unit system. The finite-element calculation model is shown as Figure 2. The simulation air area is 60 cm × 12 cm, and the grid size is 0.04 cm × 0.04 cm. Initial condition is applied to the air area and the initial specific internal energy of air is $2.068\times 10^{-3}{\mathrm{cm}}^{2}/{\mu s}^{2}$. “Flow out (Euler)” boundary is applied to the air area, which means that detonation products will not affect the formation of EFPs after flowing out of the air area. “Transmit” boundary is applied to the upper boundary of the steel target to avoid the influence of reflected stress wave on the penetration process of EFPs. Euler algorithm is used to calculate the large deformation process of liners, wave shaper and explosives. The steel target adopts the Lagrangian algorithm and applies Erosion algorithm to the target material to ensure the penetration and perforation of EFPs.

PBX (polymer bonded explosive), RDX (hexogen, cyclotrimethylenetrinitramine) and COMP-B (hexogen-trotyl composite explosive) are used for the high-detonation-velocity explosive, TNT (trotyl, trinitrotoluene) is used for the low-detonation-velocity explosive, and the wave shaper material is epoxy resin. The Lee-Tarver equation is also called the ignition growth model, the JWL (Jones-Wilkins-Lee) equation of state is used for both unreacted explosives and detonation products in the Lee-Tarver equation [29,30]. The JWL equation is expressed as Equation (5).

(5) $$P=A\left(1-\frac{\omega }{R_{1}\eta }\right)exp\left(-R_{1}\eta \right)+B\left(1-\frac{\omega }{R_{2}\eta }\right)exp\left(-R_{2}\eta \right)+\frac{\omega e}{\eta }$$

≈ P is pressure. $A,\;B,\;C,\;R_{1},\;R_{2},$ and ω are the constants related to explosives, and they are mainly obtained by fitting the cylinder test data. η is the relative specific volume, $\eta =\rho /\rho_{0}$, $\rho_{0}$ and ρ respectively represents the density of explosives before and after detonating. e is the initial internal energy per unit volume of explosives.

The ignition growth model hypothesizes that the reaction rate of explosives is controlled by the shock pressure and the surface area of hot spots formed. The reaction rate equation is divided into three terms, the first term represents the formation of hot spots, the second term represents the rapid growth process of the reaction, the third term represents the final process of chemical energy release and it is a relatively slow diffusion reaction.

(6) $$\frac{dF}{dt}=I{\left(1-F\right)}^{b}{\left(\rho /\rho_{0}-1-a\right)}^{x}+G_{1}{\left(1-F\right)}^{c}F^{d}p^{y}+G_{2}{\left(1-F\right)}^{e}F^{g}p^{z}$$

where F is the degree of explosive reaction. When F=0, it means that the explosive has not reacted and When *F* = 1, it means that the explosive has detonated. a determines the critical degree of compression, when the critical pressure is less than a, explosives cannot be ignited. y is the pressure index, in most cases y=1. b and c are burnup index. The first ignition term is a function of the intensity and duration of pressure, in this term, I and x determine the number of ignition hot spots. $G_{1}$ and d determine the duration of hot spots. $G_{2}$ and z determine the reaction rate under high pressure. The parameters of explosives [1,14,15] are shown in Table 1 and Table 2.

The outer liner is copper, the inner liner is aluminum, the target is 4340 steel. The state equation of materials is the Shock-Eos and the strength model is the Johnson-Cook. The Shock-Eos describes the relationship between pressure and internal energy at a point outside Hugoniot-curve and a point on the curve,

(7) $$P=P_{H}+\rho \Gamma \left(e-e_{H}\right)$$

where Γ is the Gruneisen coefficient, ρ is the density, $P_{H}$ is the pressure on the Hugoniot-curve. It is assumed ρΓ = $\rho_{0}\Gamma_{0}$ = constant and Γ is often approximated to 2. The Johnson-Cook equation is divided into three items, which respectively reflect the strain effect, strain rate effect and thermal softening effect of materials,

(8) $$\sigma_{y}=\left(A+B\varepsilon_{p}^{n}\right)\left(1+C\;log\;\varepsilon_{p}^{*}\right)\left(1-T_{H}^{m}\right)$$

where $\sigma_{y}$ is dynamic yield stress, $\varepsilon_{p}$ is effective plastic strain, $\varepsilon_{p}^{*}$ is normalized effective plastic strain rate, A is static yield stress, B is hardening constant, C is strain rate constant, n is hardening exponent, m is thermal softening exponent, $T_{H}$ is relative temperature, $T_{m}$ and $T_{r}$ is the melting temperature and room temperature respectively, $T_{H}=\left(T-T_{r}\right)/\left(T_{m}-T_{r}\right)$. G is shear modulus. The parameters of metal materials [31] are shown in Table 3. The wave shaper material is epoxy resin, its equation of state is Puff, and the main parameters are density $\rho =1.84\;g/cm^{3}$, Gruneisen coefficient is 0.15, and the expansion coefficient is 0.25.

### EFP Forming Process in OSC and DLSC

**Figure 3:**
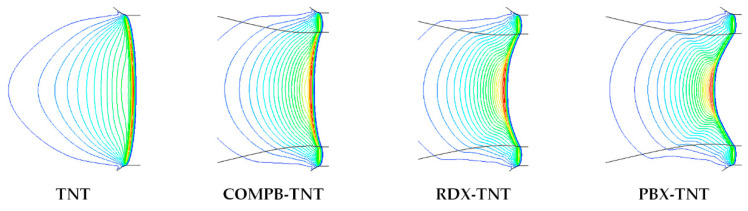
Stable propagation forms of detonation waves in OSC and DLSC.

The collision of detonation waves at the axis will produce overdriven detonation. The overdriven detonation wave will eventually decay into a steady spherical wave in an OSC. The overdriven detonation wave in a DLSC propagates as a stable horn wave, and its axis can always maintain in the overdriven detonation state. In a DLSC, the greater the differences between the detonation velocities of tow explosives, the greater the intensity of the overdriven detonation wave. Figure 3 shows the stable propagation form of detonation waves in different charge structures.

Numerical simulations are carried out on the forming process of EFP in DLSC and OSC respectively. The outer liner is copper and the inner liner is aluminum. In the DLSC, the outer layers are COMPB, RDX, and PBX in descending order of detonation velocity, and the inner layer is TNT. The OSC is TNT and RDX respectively.

**Figure 4:**
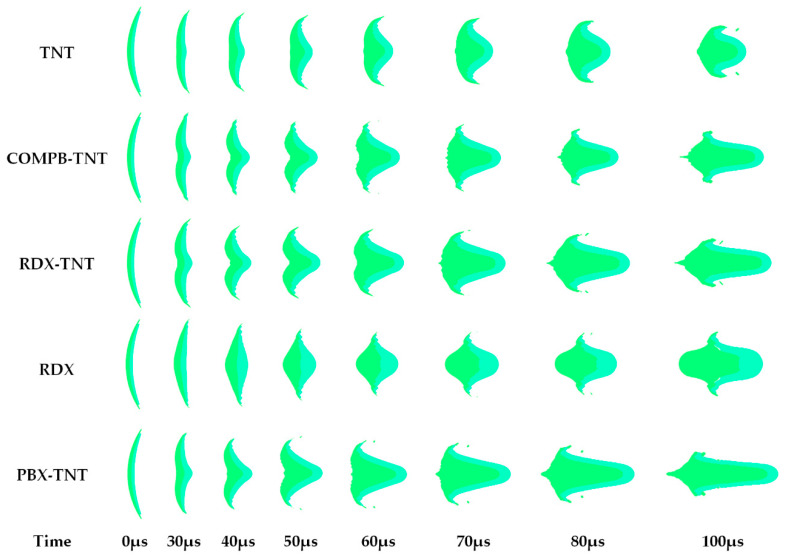
EFP forming processes in different shaped charge structures.

As shown in Figure 4, in the OSC (TNT), the attenuation rate of overdriven detonation wave intensity is greater than that of the DLSC. The EFP is ultimately a quasi-spherical shape with poor aerodynamic shape. In DLSCs, due to the overdriven detonation at the charge axis, the velocity of the liner top micro-elements is significantly higher than that of the bottom micro-elements, and the incident angle of detonation wave on the liner surface is smaller. The whole liner is collapsed to the axis, and the projectile is elongated. The greater the difference between the detonation velocity of the two explosives, the greater the aspect ratio of the projectile is. At 100 μs, a long rod EFP with a large length-diameter ratio is formed. In the OSC (RDX), the liner is collapsed into a similar “W” shape in the initial stage of forming process. Unlike other charge structures, the shape of the EFP is finally pressed forward. The three kinds of charge structures of OSC (TNT), DLSC (RDX-TNT) and OSC (RDX) are further studied, and the propagation of detonation waves in the charge and the collapsed process of liner are analyzed.

**Figure 5:**
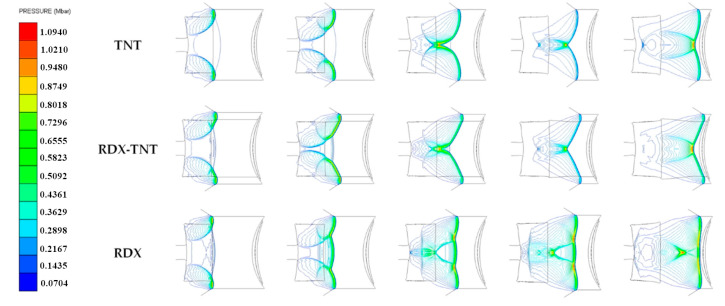
Propagation process of detonation waves in three shaped charge structures: OSC (TNT); DLSC (RDX-TNT); OSC (RDX).

As shown in Figure 5, in OSC (TNT) and DLSC (RDX-TNT), TNT as a low-sensitivity explosive, the detonation wave transmitted through the wave shaper does not reach the shock initiation threshold of TNT. The explosive at the axis will not be detonated by the transmitted wave. After detonation waves are diffracted by the wave shaper, they can collide with each other at the axis to form overdriven detonation. However, the overdriven detonation wave of the OSC (TNT) decays faster compared with the DLSC (RDX-TNT). The intensity of overdriven detonation at the axis is smaller and the incident angle on liner surface is larger. In the DLSC (RDX-TNT), the initial pressure acting on the liner top micro-element is relatively large, and the projectile is elongated to form an EFP with a large length-diameter ratio. In the OSC (RDX), RDX is a high-energy sensitive explosive, and the explosive at the axis is detonated by transmitted waves. The detonation waves at the axis interact with the diffracted detonation waves diffracting through the wave shaper. Finally, the shape of the detonation wave that acts on the liner surface is a plane wave. This situation corresponds to placing a set of electronic components inside the wave shaper of terminal-sensitive projectile, which will cause the wave shaper to be no longer uniform and dense, and the wave shaper cannot completely block the propagation of detonation waves at the axis. For DLSCs, because of the differences in detonation velocity between the two explosives, the horn waves can be formed inside the charge regardless of the performance of wave shapers.

## 4. Experimental Verification and Discussion

**Figure 6:**
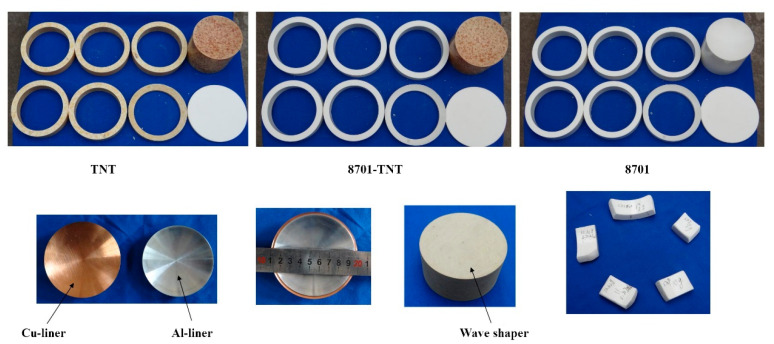
Compositions of three shaped charges: OSC (TNT), DLSC (8701-TNT) and OSC (8701).

As shown in Figure 6, the caliber of the shaped charge is 90 mm. Parameters of 8701 explosive and RDX explosive used in the numerical simulation are similar. In the experimental, the HE is 8701 and the LE is TNT. Because the outer layer of the shaped charge is annular structure, in order to ensure the uniform density of the explosive grain during the manufacturing process, the outer grain is divided into 6 rings for production. Break one of the rings, measure the density separately, and observe whether the density of the ring explosive grain is uniform everywhere. The wave shaper is epoxy resin.

**Figure 7:**
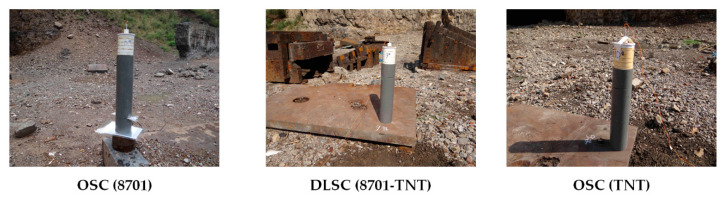
EFP penetration experimental layout of three shaped charges: OSC (TNT), DLSC (8701-TNT) and OSC (8701).

The penetration ability of EFP is verified by experiments, and the blasting height is 500 mm. The target of EFP penetration is initially set as a two-layer 4340 steel ingot with a diameter of 160 mm and a thickness of 70 mm + 30 mm. During the experimental of the OSC (8701), because the diameter of the prepared 4340 steel ingot was too small, the steel ingot was cracked after penetration. In the subsequent experiments of DLSC (8701-TNT) and OSC (TNT), the penetration target was replaced with a rolled homogeneous armour (RHA) target with a thickness of 80 mm. The main parameters of RHA are as follows: density $\rho =7.86\;g/cm^{3}$, shear modulus G=64.1 GPa, and yield stress $Y_{s}=1.5\;\mathrm{GPa}$. The experimental layout is shown in Figure 7.

**Figure 8:**
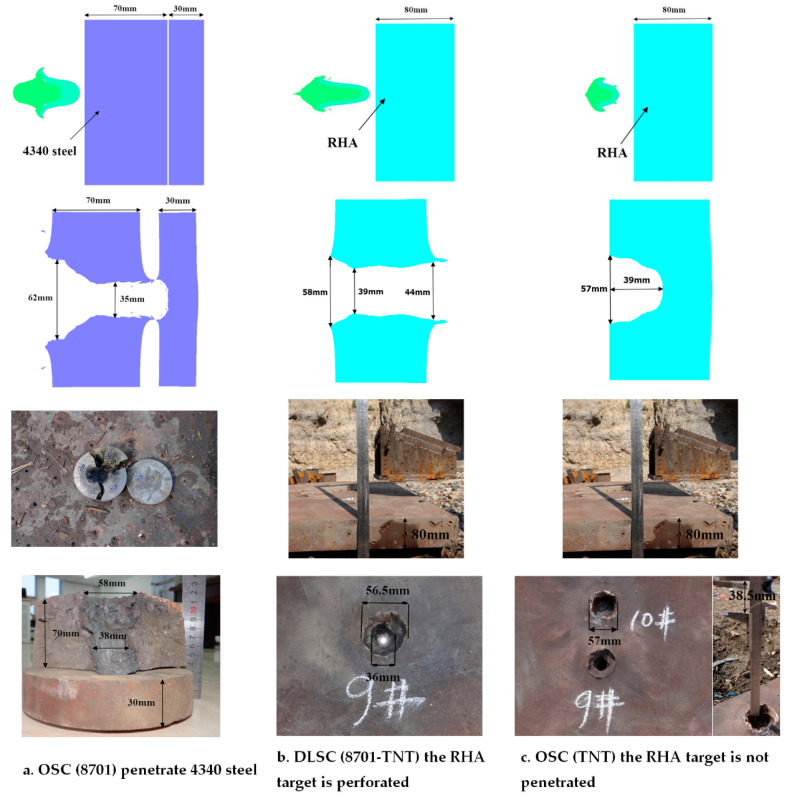
EFP penetration experimental results of three shaped charges: OSC (TNT), DLSC (8701-TNT) and OSC (8701).

The EFP penetration experimental comparisons of the three shaped charges are shown in Figure 8. The target of OSC (8701) penetration is 4340 steel with a penetration depth of 70 mm, and failed to penetrate the second layer of steel ingots with a thickness of 30 mm. The opening diameter is 58 mm. Because the diameter of the steel ingot is too small, the edge is broken. Because the diameter of the prepared 4340 steel ingot was too small, the steel ingot was cracked after penetration. The target of DLSC (8701-TNT) and OSC (TNT) penetration is RHA. DLSC (8701-TNT) can penetrate the RHA target with a thickness of 80 mm and the opening diameter is 56.5 mm. OSC (TNT) fails to penetrate the RHA target and the penetration depth is 38.5 mm. Compared with OSC (TNT), the penetration depth of DLSC (8701-TNT) is increased by at least 107.8%. The material hardness and strength of RHA are greater than 4340 steel. From the above results, it can be seen that the penetration capability of EFP formed by DLSC (8701-TNT) is greater than that of OSC (8701) and OSC (TNT). The experimental results are consistent with the numerical simulation results, which prove the accuracy of the research on the EFP formation and penetration capability in this article.

## 5. Conclusions

Detection electronic components of a small-caliber terminal sensitive projectile that are limited by space are often placed inside wave shaper, which will cause the wave shaper to no longer be uniform and dense, and weaken the ability to adjust detonation waves. In this article, we designed a DLSC with HE in the outer layer and LE in the inner layer. The propagation process of detonation waves in OSC and DLSC are compared and analyzed.

(1) In DLSCs, because of the differences in detonation velocity between the two explosives, horn waves can be formed inside the charge regardless of the performance of wave shapers. Detonation waveforms can be optimized and form overdriven detonation at the charge axis, and then a long rod EFP with a large length-diameter ratio is obtained.
(2) We designed three kinds of shaped charges to experiment with and verify the penetration capability of EFP, and they are OSC (8701), DLSC (8701-TNT), and OSC (TNT), respectively. The results show that the penetration capability of a long rod EFP with a large length-diameter ratio obtained by the DLSC (8701-TNT) is the best, and the penetration depth is increased by at least 107.8% compared with the OSC (TNT). DLSCs can optimize the detonation waveform to obtain EFPs with a better aerodynamic shape, which improves the penetration capability of EFPs for long-distance armored targets. The research results can provide a reference for the application of the DLSC in engineering and a new idea for the structure design of small-caliber terminal sensitive projectile.

## Figures and Tables

**Table 1:** JWL parameters of explosives (PBX, RDX, COMP-B and TNT).

Parameters	Unreacted Explosive				Detonation Products			
Explosive	TNT	PBX	RDX	COMP-B	TNT	PBX	RDX	COMP-B
$\rho \;\left(g\cdot cm^{-3}\right)$	1.63	1.842	1.836	1.717	-		-	-
$D\;\left(m\cdot s^{-1}\right)$	-	-	-	-	6930	8800	8239	7980
P (GPa)	-	-	-	-	21	37	32.5	29.5
A (GPa)	1798	9.52 × 10^5^	2.01 × 10^5^	7.78 × 10^4^	371.2	852.4	801.8	542.2
B (GPa)	−93.1	−5.94	−5.2	−5.0	3.23	18.02	52.64	7.67
$R_{1}$	6.2	14.1	12.4	11.3	4.15	4.6	5	4.2
$R_{2}$	3.1	1.41	1.24	1.13	0.95	1.3	2.1	1.1
ω	0.8926	0.8867	0.8867	0.8938	0.3	0.38	0.34	0.34
$e\left(J/mm^{3}\right)$	7.0	10.2	8.5	8.1	-	-	-	-

**Table 2:** Reaction rate parameters of explosives (PBX, RDX, COMP-B and TNT).

Parameters	TNT	PBX	RDX	COMP-B	Parameters	TNT	PBX	RDX	COMP-B
$I\left(\mu s^{-1}\right)$	50	0	14	44	d	0	0	0	0.667
b	0.667	0.667	0.667	0.667	y	0	3.2	0	2
a	0	0	0	0.01	$G_{2}$	40	24	40	0
x	4	0	4	4	e	0.222	1	0.222	0
$G_{1}$	0	1899	488	414	g	0.666	1	0.666	0
c	0.667	1	0.667	0.222	z	1.2	1	1.2	0

**Table 3:** Parameters of metal materials.

Materials	$\rho \left(g/cm^{3}\right)$	Γ	G(GPa)	A(GPa)	B(GPa)	n	c	m	$T_{m}\left(K\right)$
Copper	8.96	2.00	46	0.09	0.292	0.31	0.025	1.09	1356
Aluminum	2.70	1.97	27.1	0.04	0.48	0.27	0.01	1.00	1220
4340 Steel	7.83	2.17	81.8	0.792	0.51	0.26	0.014	1.03	1793

## Abbreviations

DLSC	Double-layer shaped charge
OSC	Ordinary shaped charge
EFP	Explosively formed projectiles
HE	High-detonation-velocity explosive
LE	Low-detonation-velocity explosive
SCWS	Shaped charge with wave shaper
