# Correction: Liu et al. The EFP Formation and Penetration Capability of Double-Layer Shaped Charge with Wave Shaper. *Materials* 2020, *13*, 4519

**DOI:** 10.3390/ma14092210

**Published:** 2021-04-25

**Authors:** Yakun Liu, Jianping Yin, Zhijun Wang, Xuepeng Zhang, Guangjian Bi

**Affiliations:** School of Mechatronic Engineering, North University of China, Taiyuan 030051, China; lykcpy@163.com (Y.L.); wzj@nuc.edu.cn (Z.W.); zhangxp@nuc.edu.cn (X.Z.); bgjnuc@163.com (G.B.)

The authors wish to make the following corrections to this paper [1]:

Figure 1, Equations (1)–(3) in our article.

According to our previous researches [2,27], in a DLSC, detonation waves that propagate in the LE are no longer centered at the $O^{\prime }$ point and the center of the detonation waves becomes the O point. The incident angle of detonation wave in the horizontal direction of shaped charge is $\varphi_{DLSC}$,

(1) $$\varphi_{DLSC}=arcsin\left\{\frac{\left[n^{2}+{\left(D_{L}r^{i}\right)}^{2}-2D_{H-L}n\sqrt{l^{2}+r^{i}{}^{2}}+2D_{L}ln\right]}{{\left(D_{L}r^{i}\right)}^{2}+n^{2}}\right\}$$

in the Equation (1), $n=D_{H-L}\sqrt{l^{2}+r^{i}{}^{2}}-D_{L}l$, $r^{i}=r_{2}-y^{i}$. $D_{L}$ is the detonation velocity of LE, $D_{H-L}$ is the propagation velocity of detonation wave when the LE is shocked initiation by the HE near the contact surface of two explosives. u is the particle velocity of explosive. The subscripts H and L represent the HE and LE respectively.

(2) $$D_{H-L}=D_{L}\left(\frac{u_{H}}{u_{L}}+\frac{u_{L}}{u_{H}}\right)/2\;,\;u=\frac{1}{k+1}D$$

From the geometric relationship in Figure 1, the value of $\varphi_{E}$ can be obtained.

(3) $$\{\begin{array}{c} \varphi_{E}=\varphi_{DLSC}-\alpha ;y^{i}<r_{2}\;\\ \varphi_{E}=90-\alpha +\theta ,\theta =arctan\left(\frac{y^{i}-r_{2}}{x^{i}+l}\right);y^{i}>r_{2} \end{array}$$

where α is the equivalent half cone angle. φ is the incidence angle of detonation waves on the surface of micro-elements.

In the subsequent research process, we found that there was no need to calculate the values of $D_{H-L}$ and $\varphi_{DLSC}$, so the contents of the Figure 1 should be modified, Equations (1)–(3) should be replaced. The content of “φ is the incidence angle of detonation waves on the surface of micro-elements” should be changed to “$\varphi_{E}$ is the incidence angle of detonation waves on the surface of micro-elements”.

For the calculation of the incident angle of the detonation wave, when $\frac{\pi }{2}-\alpha <\gamma$, it can be obtained from the geometric relationship shown in the figure: (where *R* is the curvature radius of the liner):

(1) $$\varphi_{E}=\gamma -\left(\frac{\pi }{2}-\alpha \right)+acos\frac{{\left[R-Rcos\left(\frac{\pi }{2}-\alpha \right)+l\right]}^{2}+{\left[Rsin\left(\frac{\pi }{2}-\alpha \right)-r_{2}\right]}^{2}+{\left(R+l\right)}^{2}+r_{2}{}^{2}-R^{2}}{2\sqrt{{\left[R-Rcos\left(\frac{\pi }{2}-\alpha \right)+l\right]}^{2}+{\left[Rsin\left(\frac{\pi }{2}-\alpha \right)-r_{2}\right]}^{2}}\sqrt{{\left(R+l\right)}^{2}+r_{2}{}^{2}}}$$

When $\frac{\pi }{2}-\alpha >\gamma$, it can be obtained:

(2) $$\varphi_{E}=\frac{\pi }{2}-\alpha -\gamma +acos\frac{{\left[R-Rcos\left(\frac{\pi }{2}-\alpha \right)+l\right]}^{2}+{\left[Rsin\left(\frac{\pi }{2}-\alpha \right)-r_{2}\right]}^{2}+{\left(R+l\right)}^{2}+r_{2}{}^{2}-R^{2}}{2\sqrt{{\left[R-Rcos\left(\frac{\pi }{2}-\alpha \right)+l\right]}^{2}+{\left[Rsin\left(\frac{\pi }{2}-\alpha \right)-r_{2}\right]}^{2}}\sqrt{{\left(R+l\right)}^{2}+r_{2}{}^{2}}}$$

From the geometric relationship in Figure 1, the value of γ can be obtained.

(3) $$\gamma =arctan\left(\frac{r_{2}}{l+R}\right)$$

where α is the equivalent half cone angle. $\varphi_{E}$ is the incidence angle of detonation waves on the surface of micro-elements.

Since this part only introduces the collapsing process of the liner in the figure, it has not been theoretically calculated and this part has no influence on the main research ideas and contents of the paper. The modification in this section does not affect the subsequent numerical simulation analysis and experimental results.

We apologize for the inconvenience caused to the readers by our mistakes.

## Figures and Tables

**Figure 1:**
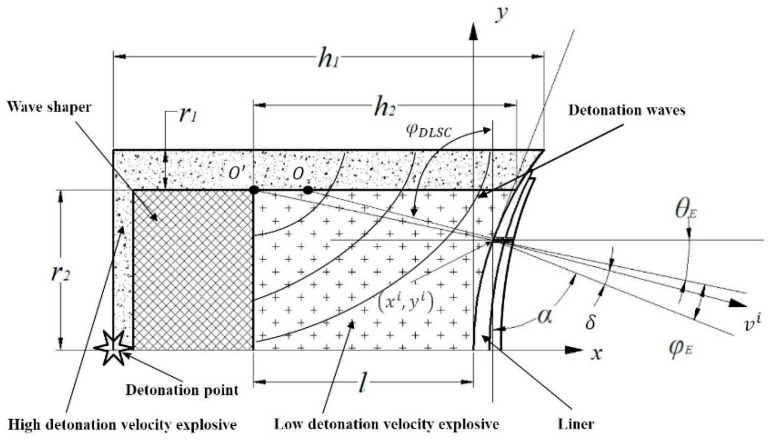
Detonation wave propagation in DLSC.

**Figure 1:**
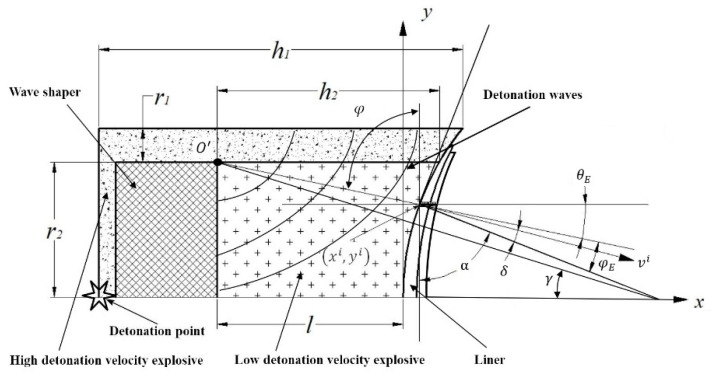
Detonation wave propagation in DLSC.
